# Pellagra and War Diet

**Published:** 1919-02

**Authors:** J. R. Lowrey

**Affiliations:** Raleigh, N. C.


### Pellagra and War Diet.

DR. J. R. LOWREY, RALEIGH, N. C.

Since Pellagra was first recognized in America, every physician that has treated any number of cases has advanced a theory as to its cause. But so far none of them have proven any theory to be correct. We are just as far from the real cause as we were ten years ago, when it first appeared in sufficient numbers in this country to be recognized. The maize theory has long been cast aside by practically every one that has studied the disease at all.

The nitrogenous diet theory advocated by Dr. Goldberger is the most prominent theory that has been advocated of recent years due to the prominence of the man and the Public Health Service which he represents. But this world war has demonstrated to the medical profession that the lack of a well balanced diet being the cause of pellagra is fallacious.

At no time in the history of the disease have we suffered from such an insufficient quantity of introgenous food, the very food, that Dr. Goldberger says the lack of, causes Pellagra, as we have during this war and the number of cases of the disease has not increased one bit. I find this true in my practice, and those physicians that I have talked with from overseas report the same thing on the other side.

Dr. Goldberger is a good man and honest in his belief that Pellagra is due to an unbalanced diet, but he should have experimented further and on a greater number of cases before he advocated an unbalanced diet as the cause of this disease and a nitrogenous diet alone as a cure for it.

If eating an unbalanced diet caused Pellagra, Serbia, Belgium, and Mexico, might easily be annihilated from its ravages instead of by bullets. So, too, would it have been rampant during the Civil War and during drouths, where corn syrup and bread was the principal diet.

Who would dare say that an unbalanced diet caused Pellagra when the largest per cent of the world has been living on an unbalanced diet for two years and Pellagra has not even been mentioned once as being one of the results of the war.

The purpose of this paper is not only to show the part the war has played in this disease but also to bring before the medical profession the great harm that has been done by trying to cure Pellagra by diet alone. A number of cases can be cured by diet alone, so can a number of cases of any curable disease, but that is no reason why we should discard all drugs in treating those diseases. You take a number of cases of Malaria and force feed them on a strong nitrogenous diet and many of them will recover without any other treatment, but he who would attempt to treat Malaria as a general routine by diet alone should be ostracized from the medical fraternity. In typhoid fever, nephritis, diarrhoea, and tuberculosis as well as many other diseases, more can be done toward recovery by diet than can be accomplished by medicine. But that is not evidence of the fact that these diseases are the result of an unbalanced diet.

I have seen patients who were suffering from the severest form of Pellagra with the eruption on the hands, arms, face, and neck, with the other constitutional symptoms, that were fat, young and strong. I have seen these same people get well and they are well today, by having been given the arsenic treatment, not one word being said to them about the diet. They are eating the same kind of diet that they have been eating for the past twenty years.

Dr. J. H. Graves of Waco, Texas, has advanced a most plausible theory: He says pellagra is due to the mosquito Culex and the aedea calopus. These mosquitos breed in filth, and especially sewer filth. We know that we have more pellagra in filthy places and where there is poor sewerage. We also know when we clean up the sewers we have less Pellagra. We have pellagrins in greater numbers in the mosquito section of this country, namely, the South, than elsewhere. Joblin and Peterson carried on a survey for two years in the city of Nashville, and the following facts were established, according to their report: They say that a definite number of cases developed in individuals partaking of a diet as varied and as wholesome as could be demanded by any advocate of the dietary theory, and that cases of Pellagra developed in the breast fed children of nonpellagrous mothers.

Arsenic is just as antagonistic to Pellagra as antitoxin is to diphtheria. I do not mean that every physician who treats Pellagra with arsenic gets as good results as he does with diphtheria antitoxin, but I do claim that if he will give it in the vein and in proper doses that it will result in as many cures as antitoxin does in diphtheria.

Every physician that has used arsenic in any form for this dread disease knows that he gets more or less good results by giving it, but those who give it in the veins know that the results are marvelous and it seems a pity that such a prominent man as Dr. Goldberger should advocate the discard of all drugs and treatment other than diet.

I have treated all the Pellagra patients at the North Carolina Confederate Soldiers' Home, for the past five years. We average about five patients a year with Pellagra. They all eat the same diet. If the disease is due to diet why is it that the other 150 inmates do not develop the disease. During this four years there has not been a death due to Pellagra. All have been treated with arsenic and the diet has not been changed.

During last summer I treated a number of Pellagra patients from the country that lived on a farm and have been eating the same kind of diet for the past twenty years. I gave them all arsenic in the vein and they all recovered. What they should eat was not mentioned. If they had asked what they should eat I would have told them anything and everything that they wanted and could get.

